# Historical ecology with real numbers: past and present extent and biomass of an imperilled estuarine habitat

**DOI:** 10.1098/rspb.2012.0313

**Published:** 2012-06-13

**Authors:** Philine S. E. Zu Ermgassen, Mark D. Spalding, Brady Blake, Loren D. Coen, Brett Dumbauld, Steve Geiger, Jonathan H. Grabowski, Raymond Grizzle, Mark Luckenbach, Kay McGraw, William Rodney, Jennifer L. Ruesink, Sean P. Powers, Robert Brumbaugh

**Affiliations:** 1 Department of Zoology, University of Cambridge, Cambridge CB2 3EJ, UK; 2 Global Marine Team, The Nature Conservancy, Department of Zoology, University of Cambridge, Cambridge CB2 3EJ, UK; 3 Washington State Department of Fish and Wildlife, Point Whitney Shellfish Laboratory, 1000 Point Whitney Road, Brinnon, WA 98320, USA; 4 Department of Biological Sciences, Florida Atlantic University, c/o Harbor Branch Oceanography Institute, 5775 Old Dixie Highway, Fort Pierce, FL 34946, USA; 5 USDA, Agricultural Research Service, Hatfield Marine Science Center, 2030 SE Marine Science Drive, Newport, OR 97365, USA; 6 Fish and Wildlife Research Institute, Florida Fish and Wildlife Conservation Commission, 100 Eighth Avenue SE, St Petersburg, FL 33701, USA; 7 Marine Science Center, Northeastern University, 430 Nahant Road, Nahant, MA 01908, USA; 8 Department of Biological Sciences, University of New Hampshire, Durham, NH 03824, USA; 9 Virginia Institute of Marine Science, College of William and Mary, PO Box 1346, Gloucester Point, VA 23062, USA; 10 NOAA Restoration Center, 1315 East West Highway, Silver Spring, MD 20910, USA; 11 Texas Parks and Wildlife Department, Dickinson Marine Laboratory, 1502 FM 517 East, Dickinson, TX 77539, USA; 12 Department of Biology, University of Washington, Box 351800, Seattle, WA 98195-1800, USA; 13 Department of Marine Sciences, University of South Alabama, Dauphin Island Sea Laboratory 101 Bienville Boulevard, Dauphin Island, AL 36528, USA; 14 The Nature Conservancy, 127 Industrial Drive, Big PineKey, FL 33042, USA

**Keywords:** shifting baseline, *Crassostrea virginica*, *Ostrea lurida*, native oyster, United States

## Abstract

Historic baselines are important in developing our understanding of ecosystems in the face of rapid global change. While a number of studies have sought to determine changes in extent of exploited habitats over historic timescales, few have quantified such changes prior to late twentieth century baselines. Here, we present, to our knowledge, the first ever large-scale quantitative assessment of the extent and biomass of marine habitat-forming species over a 100-year time frame. We examined records of wild native oyster abundance in the United States from a historic, yet already exploited, baseline between 1878 and 1935 (predominantly 1885–1915), and a current baseline between 1968 and 2010 (predominantly 2000–2010). We quantified the extent of oyster grounds in 39 estuaries historically and 51 estuaries from recent times. Data from 24 estuaries allowed comparison of historic to present extent and biomass. We found evidence for a 64 per cent decline in the spatial extent of oyster habitat and an 88 per cent decline in oyster biomass over time. The difference between these two numbers illustrates that current areal extent measures may be masking significant loss of habitat through degradation.

## 1. Introduction

Humans have been modifying ecosystems and exploiting natural populations for millennia [1]; however, quantitative data on the impacts of our exploitation over large spatial scales, whether terrestrial or marine, are primarily limited to recent decades [2–4]. Even over this short time frame, many populations and habitats have undergone unprecedented change [5–7]. In the heavily modified ecosystems existing today, an understanding of historical conditions can provide a robust baseline for assessing change, modelling past ecosystem functions, assessing the need for conservation interventions, setting realistic restoration goals, planning restoration activities, and critically, for guiding management practices in the face of global change [8]. To these ends, improved methods for understanding the status and functioning of ecosystems prior to or during the early stages of anthropogenic impacts are needed.

In terrestrial settings, modelled potential vegetation maps are a widely used proxy for describing historic or original vegetation cover [9], although such maps cannot account for all variables, nor for the gradual and partial human modification of landscapes over millennial timescales [10]. Such predictive approaches are even more challenging in marine and coastal environments, where poor understanding of driving variables and lack of data still prevent any reliable prediction of habitat distribution at large scales [11]. Historic baselines in the marine environment must therefore be pieced together using historical records of species, fisheries data, navigational maps and charts, and naturalists’ descriptions. Recent studies have drawn on a wide range of such anecdotal and semi-quantitative historical evidence to draw a compelling picture of local to regional changes in marine and coastal environments [12–15]. While such works greatly enhance our understanding of historic conditions, they remain limited in their capacity to quantify change.

Detailed quantification of change is dependent on large-scale datasets. For a few habitat types, such information can be found in early land registries and charts [16,17], however, most marine habitats remained poorly documented until the mid to late twentieth century and the widespread availability of remote sensing technologies [4,18–20]. As a result, assessments of change in many marine habitats and populations are sensitive to shifting baselines [21,22]. Oyster grounds in the United States are a valuable exception to this data paucity in marine habitats, having been surveyed as early as 1878 [23].

Habitat-forming oysters are an ecologically important and historically dominant feature of North American estuaries [24,25], where they have significant cultural and economic value [26]. Two species within the family Ostreidae dominate: *Crassostrea virginica* (Gmelin 1791), the eastern oyster on the Atlantic and Gulf coasts, and *Ostrea lurida* Carpenter 1864, the Olympia oyster on the Pacific coast. In unmodified conditions, both have the capacity to build large reefs or beds—physical structures with a veneer of living oysters overgrowing non-living shell deposits of prior oyster generations. Such biogenic habitats are rich in associated species and offer a range of ecosystem service benefits, including enhancing non-oyster species of commercial value, coastal protection and biofiltration of the water column [27–29].

Oysters have been fished for thousands of years [1], however, drivers such as the intensification of exploitation, changes in coastal hydrology and the impact of diseases, have led to significant declines in this valuable habitat over the past 200 years or so [12]. A number of studies have sought to estimate the decline in oyster grounds over this time period, using expert syntheses and proxy records [13,14,30]. All illustrate significant changes expressed as fisheries collapse, population decline, change in areal extent or some combination thereof. Such studies undoubtedly have a powerful influence on perceptions of the habitat and on broad policy decisions, but greater detail is needed to influence management interventions. Moreover, the reliance on fishery-dependent data (e.g. landings, fishery-related legislation) in such analyses has resulted in some scepticism regarding the magnitude and causes of the documented declines [31,32].

Our study, to our knowledge, builds the first quantitative record of the historic and present extent and biomass estimates of oyster grounds in the United States (lower 48 States; hereafter termed US). Accurate inventories of oyster grounds were and are undertaken because of their considerable economic value and perceived decline, combined with their distribution predominantly in waters under state jurisdictions. Fisheries policies have often aimed to encourage the leasing of bottom for managed oyster harvest and aquaculture, but in order to do this, it was necessary to delimit existing grounds as public resources. This necessity, coupled with an interest in determining the condition of public oyster grounds, led to a large number of federally funded oyster mapping expeditions during the late 1800s and early 1900s ([23,33], see the electronic supplementary material for a full reference list). Mapping was facilitated by the nature of oyster reefs, which form structurally distinct patches in the soft mud or sand bottom of estuaries. In addition, their structure can be clearly determined by touch or physical sampling, thereby allowing subtidal mapping at a time when visual examination of the subtidal was not possible. Many of these surveys provided both details of oyster extent and quantitative information on the density of oysters.

While historic data incorporating both density and extent measures are available for some temperate forests over relatively large scales at a similar time period [34], the only coastal habitat data we are aware of, which combine both extent and some measure of habitat condition are for the Sundarbans mangroves of Bangladesh (1926–1997) [35]—a dataset that is both more recent and less extensive than our own. As such, these historic records provide an unrivalled resource with regards to the historic condition (areal extent; mean oyster shell height (SH); density, and biomass) of this critical coastal habitat. Modern stock assessments provide a similar suite of data that consequently permit assessment of long-term changes in habitat quantity and quality.

The decline of oyster habitats in the US, coupled with growing recognition of the importance of non-fishery-related ecosystem services provided by these habitats, has been increasing in recent years [27,28,36]. This has led to significant federal- and State-level investment in oyster reef restoration. More than 10 million US dollars was directed to oyster reef restoration by the National Oceanic and Atmospheric Administration (NOAA) through the American Recovery and Reinvestment Act of 2009, roughly equivalent to the previous 10 years of oyster reef restoration funding. As ecologists and natural resource managers strive towards restoring coastal ecosystems, quantitative assessment of the historic extent and habitat quality, whether for oysters or other habitats, will provide an invaluable tool to guide and inform restoration efforts.

## 2. Material and methods

### (a) Data review

We conducted a thorough review of quantitative information on the historic and present extent and condition of oyster reefs in the US, drawing on scientific literature, historic United States fishery reports, State fisheries reports and publicly available data (see the electronic supplementary material). Such data, even historically, were the result of highly detailed surveys, typically with boat-based sampling over a period of several weeks, involving tens of full-time researchers. We summarized the findings into sub-estuarine drainage areas (sub-EDA), as listed in NOAA's coastal assessment framework (CAF) [37]. Sub-EDAs equate to whole estuaries, with the exception of Chesapeake Bay and Puget Sound, which are subdivided into their major tributaries. Hereafter, we refer to all sub-EDA units as estuaries. The relevant data on extent, SH and density were extracted and catalogued, and the number of oysters per bushel was noted in order to derive an estimate of mean SH. Bushels are volumetric measures used by fishers and fisheries managers. A legally defined standard US bushel (3.52 × 10^4^ cm^3^) is sometimes used, although more typically legal bushels are defined at the state level. If not clearly stated in the source, then we were able to infer whether a state-defined or a standard bushel was used by more detailed investigation. For example, Moore states in his 1910 survey of the James River, VA, that ‘oysters on this bed are large, averaging … . over 300 per bushel’ [38, p. 15]. Oysters would have averaged 75 mm (approximately the cut off for market size, and thus not large) using a standard bushel, or 89 mm if the Virginia bushel is applied. If there was doubt as to the bushel size, then the standard US bushel was applied, because it resulted in a more conservative estimate of SH.

### (b) Oyster extent

Universal definitions of the habitat classification allowed for a more robust assessment of change in spatial extent. The vast majority of historical and present-day oyster habitat surveys were conducted for fisheries management purposes and use a relatively consistent approach. For these cases, we used the term ‘oyster grounds’ that we define as the wider community complex of which oyster reefs and beds are clearly an important part, but that also includes areas of adjacent sediments and shell rubble. Such areas would broadly equate with ‘fishable areas’. Historically, only areas with oysters at densities high enough to support fishing activity were included in surveys; isolated individuals and groups that were not forming beds or reefs were excluded. Such thresholds are still applied in modern mapping approaches. Consequently, it is possible for the species to persist in an estuary, but for there to be no remaining oyster grounds. We term this loss of habitat as the species being functionally extinct.

Most sources provided direct numerical estimates of the extent of oyster grounds. Where only maps were available, they were digitized, and the areal extent of mapped oyster grounds was calculated using Arc Geographical Information Systems. Maps were also digitized if the areas described straddled two estuary units, such that the extent in each estuary could be determined. In a small number of cases, areal extent had been estimated instead of being directly surveyed [39]. We considered the potential resolution of side-scan sonar (a popular modern technique) to be equal to the historic survey method of marking the boundaries of oyster grounds by dragging chains and probing the ground with poles. Where extent had been estimated, it was assumed to be an estimate of oyster grounds. Where historic oyster extent was determined multiple times, the surveys using the most direct measurement techniques were favoured. Where methods did not differ, the oldest report was used.

### (c) Oyster density

Oyster density was recorded in several historic surveys undertaken towards the end of the nineteenth century and the early twentieth century. Frequently, the oyster count within size classes (typically greater than 76 mm, 76–25 mm, less than 25 mm) was documented. The majority of surveys determined oyster density by tonging a number of locations within each delineated oyster ground. A tong is a traditional harvesting tool composed of two rakes joined at approximately one-third of the length of the handles, such that oysters can be collected at depth with a scissor motion. A sample area was typically staked out, and tonged repeatedly until ‘everything on the bottom’ had been collected [40], we therefore assumed 100 per cent catch efficiency in our use of tonging data. A small number of historic and present-day datasets sampled oyster grounds using a dredge [23,41]. Dredges (a weighted frame dragged behind boats to collect shells and oysters scraped into the attached net) are an inefficient sampling gear, leaving many individuals behind in the area sampled. The percentage of the population collected in the sampled area (termed ‘dredge efficiency’) is highly variable, but frequently falls in the range of 15 per cent [42–46], and occasionally as low as 7.8 per cent in survey mode [43]. Therefore, as all but one series of dredge data used in our study were recent, dredge efficiency was assumed to be 8 per cent, so as to be conservative in our estimates of the change in oyster abundance. All dredge hauls with no oysters or those containing only spat (oysters less than 25 mm) were discounted to control for the potential that areas outside of oyster grounds had been sampled. The density of spat was not included in our study to control for seasonal variability, and inconsistency between studies in recording spat data. Where oyster density data for an estuary were absent, density data from the nearest estuary within the same ecoregion [47] were used as a proxy for density where appropriate (see the electronic supplementary material).

During the data-gathering process, every effort was made to understand the spatial scale at which density data were collected relative to areal extent. For a small number of estuaries, density data were collected at a fine spatial scale but mapping related to larger oyster ground units. In these cases, we applied a correction factor to account for the high mean densities reported. We determined that the proportion of barren ground within the area mapped as oyster grounds in Matagorda Bay, TX, by Moore [40] was 50 per cent (area-weighted mean). We used this correction factor to estimate the mapped oyster bottom area covered by oysters at the surveyed density.

The majority of our data represent subtidal oyster populations, which can have starkly different population structures from intertidal populations [48]. We therefore used only subtidal eastern oyster data when comparing mean market size and mean densities within each estuary over time.

### (d) Oyster size and biomass

Mean oyster SH was rarely noted in early surveys, however, the mean number of oysters in a bushel was occasionally stated, or could be inferred through assessment of the number of bushels attributed to an acre of known density. Hopkins [49] noted that the mean oyster size could be inferred from the number of oysters in a bushel of known volume. We therefore fitted a regression to the log data from Hopkins [49], and subsequently tested the strength of the correlation between the SH estimated from the number of oysters in a bushel or sack of known volume and the mean SH reported in five studies from a broad geographical range (*n* = 24). The linear regression fitted to log length and number of oysters per sack was highly significant (adjusted *r*^2^ = 0.93, *F*_58_ = 809, *p* < 0.0001), and yielded the following predictive relationship between the number of oysters in a known volume and the mean oyster length: *h* = 10^(−0.3537 log *b* + 2.8361)^, where *h* is SH (from umbo to growth edge) in millimetres, and *b* is the number of oysters in a 52.85 l volume (standard Louisiana sack). The estimated SH showed a near-perfect correlation with mean SH collected from the literature (Pearson's correlation coefficient = 0.94, *t*_21_ = 12.9, *p* < 0.0001), supporting our use of this equation to determine *C. virginica* SH across the US. SHs from nearest estuaries for which the data were available were used as proxies in estuaries where such data were not available.

It was frequently possible to derive the mean SH of two size classes of oysters (submarket and market) from the historic data: in these, the mean SH of the submarket oysters, termed ‘culls’, was conservative, as the number per bushel used included spat. With present-day data, we determined mean SH for the same-size categories (excluding spat) from size frequencies available from quadrat and dredge sampling undertaken by state fisheries managers. We then tested whether the SH data for market-sized oysters from this fishery independent data had changed over time (two-tailed *t*-test).

Oyster biomass scales with SH; however, the nature of that relationship varies regionally. In order to most accurately estimate the biomass of oysters in a given estuary, we collated SH to dry tissue mass conversions from 13 estuaries in seven states. Conversions were applied to the nearest estuaries within the same ecoregion.

### (e) Quantitative comparison

We found data that allowed direct comparison of historic and present oyster grounds and biomass in 24 estuaries throughout the US and calculated per cent change in extent and biomass over time. An estimate of change within ecoregions was determined by summing the extent and biomass in estuaries for which data were available in both time periods, within each ecoregion. Nationwide change was similarly assessed by summing and comparing all historic and present oyster extent and biomass.

Comparable quantitative data were available for only present or historic time periods for a large number of estuaries (*n* = 38). In order to analyse the change over time, we calculated the proportion of the estuary area (as listed in CAF), containing oyster grounds, so as to ensure that all estuaries were equally represented. In SC, where modern habitat mapping has been undertaken throughout the marsh areas and creek margins, estimates of areal extent were limited to oyster grounds within 5 m of the creek edge. All estuaries for which data were available were included in this analysis and each coast was analysed independently. Data were non-normally distributed and were compared using a Kruskal–Wallis test. All statistical tests were run in R v. 2.13.1 (2011-07-08).

## 3. Results

**Figure 1.**
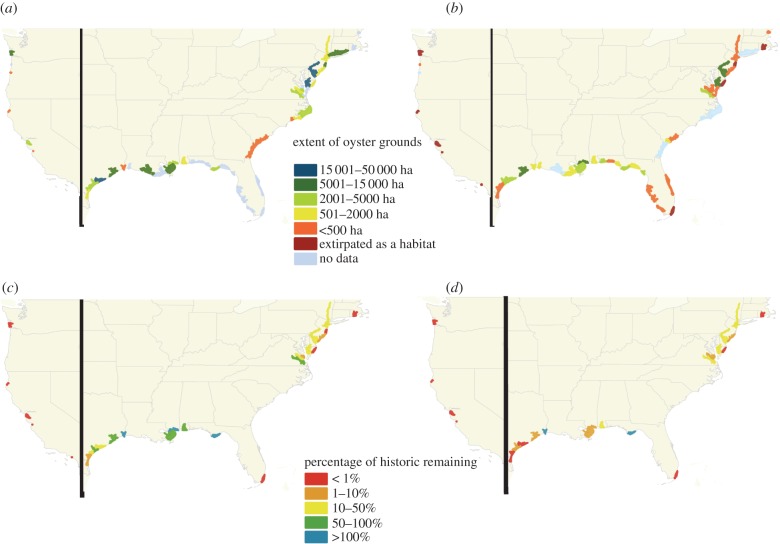
Maps illustrating: oyster ground areal extent (*a*) historically and (*b*) presently in estuaries in the US and the percentage change in (*c*) oyster ground extent and (*d*) oyster biomass in estuaries for which comparable historic and modern data were available.

**Figure 2.**
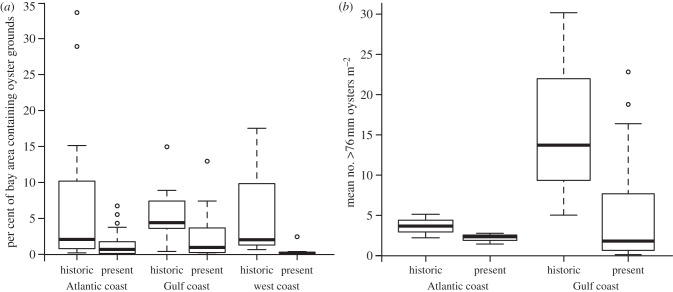
A box-whisker plot of (*a*) the percentage of estuary area containing oyster grounds past and present by coast. Proportion of estuary area occupied was significantly higher historically along all coasts (quasi-binomial generalized linear model; Kruskal–Wallis χ^2^_1_ = 5.1, *p* = 0.02; χ^2^_1_ = 5.2, *p* = 0.02; χ^2^_1_ = 8.3, *p* < 0.01 for the Atlantic, Gulf and West coasts, respectively). (*b*) The mean estuary wide density of market-sized eastern oysters historically and presently on subtidal oyster grounds in the US (Atlantic estuaries *n* = 6; Gulf estuaries *n* = 21; Kruskal–Wallis χ^2^_1_ = 2.76, *p* = 0.05).

Data on oyster extent were identified for 62 estuaries (39 historically (1878–1935, predominantly 1885–1915) and 51 estuaries more recently (1968–2011, predominantly 2000–2010); figure 1). The most extensive oyster grounds surveyed historically included: 35 536 ha in Tangier and Pocomoke Sounds (MD and VA) in 1878 on the Atlantic coast, 16 679 ha in Matagorda Bay, TX in 1907–1915 on the Gulf coast, and 6225 ha in Willapa Bay, WA in the mid-1800s on the Pacific coast (see the electronic supplementary material). The proportion of estuary area containing native oyster grounds has decreased significantly across the US (figure 2*a*).

Direct estuary-by-estuary estimates of change over time were restricted by available data to 24 estuaries, representing 16 per cent of US estuaries by number and distributed across five marine ecoregions (figure 1*c,d*). Both overall extent and biomass of oyster grounds decreased precipitously (by 64% and 88%, respectively). Losses occurred in all ecoregions for both the extent and the estimated total biomass of oysters in oyster grounds (figure 1*c*,*d*). The Olympia oyster habitat on the west coast was recorded as functionally extinct in all estuaries for which data were available for comparison. Indeed, the current 4 ha of oyster habitat recorded in Netarts Bay, OR, is the result of recent and ongoing restoration work, and has yet to form a self-sustaining population [50]. It should, however, be noted that Puget Sound, WA, contains some apparently healthy US Olympia oyster beds but was not represented in this assessment owing to a lack of estuary scale data.

The most dramatic losses of eastern oyster habitat were recorded from the northeastern Atlantic coast, with less than 6 per cent of historic extent remaining in half of the 10 estuaries where data were available (figure 1*c*). Similarly, losses in biomass were evident in the Gulf of Mexico west of the Mississippi River (figure 1*d*). It is worth noting that not all estuaries have suffered decline in either oyster extent or biomass since our approximate 1900 baseline; two estuaries (Apalachicola Bay, FL; Sabine Lake, TX and LA) showed stable or even increasing extent and biomass on oyster grounds (figure 1*c*,*d*).

Across estuaries with size and density data, we found no significant difference in mean market eastern oyster size (greater than 76 mm) over time (two sample *t*-test, *t*_17.69_ = −1.08, *p* = 0.29), while the mean market-size eastern oyster density showed a non-significant trend towards lower densities over time (figure 2*b*). The median density of subtidal market size eastern oysters declined from five to two oysters per square metre nationally and from 14 to 2 oysters m^−2^ in the Gulf of Mexico.

While the overall percentage loss in oyster biomass is greater than the change in extent, this number hides some important regional variation. Excluding estuaries where oysters are deemed functionally extinct, the biomass and extent changes are closely allied in 10 of the 18 estuaries, but the remaining eight estuaries, all in the northern Gulf of Mexico, show a decline in biomass over three times greater than the decline in oyster reef extent (figure 1*c*,*d*). This substantial decline is primarily a consequence of declines in oyster density (see the electronic supplementary material).

## 4. Discussion

The disappearance of previously productive oyster grounds was noted as far back as 1658 [12]. Scientists in the US were able to draw on extensive documentation of the decline of the European oyster species, *Ostrea edulis* Linnaeus, 1758 in Europe, to express their concerns for both commercially important North American species throughout the 1800s [51]. Today, the European oyster is considered to be functionally extinct throughout much of its range [52]. Our findings suggest that despite more than 130 years of science and calls for conservation interventions in state and federal fisheries reports and in peer-reviewed literature, both the Olympia oyster and the eastern oyster appear to have followed suit in portions of their range. Clearly, the greatest declines in oyster grounds have been along the Pacific coast, where our data reflect what is widely agreed to be a regional trend of functional extirpation of native oysters. Declines have also been considerable along the northeastern portion of the Virginian ecoregion, where two-thirds of historic extent and biomass have been lost since the late 1800s alone (figure 1*c*,*d* and the electronic supplementary material).

All previous studies that illustrated collapse or decline in oyster extent drew on fisheries data [12–14,30,53]. Those studies therefore either make no attempt to quantify loss [12,13], or quantify loss through proxies (landings data) sometimes combined with delphic processes [14,30,53], resulting in high uncertainty [54]. By relying on fisheries-independent data, we seek to end the debate surrounding the extent of decline in oyster habitat in US estuaries [31,32,54]. It must, however, be stressed that despite the relative robustness of our historic dataset, our study does not reflect the decline from pristine baselines. For most estuaries assessed, the historic quantitative baseline was measured at a point in time when the estuaries were already impacted by fishing. Indeed, the major impetus for surveying the grounds historically was a perceived vulnerability or observed declines in natural oyster resources, with the declines frequently linked to overexploitation [33,55,56]. A review of the historic literature illustrates that such overexploitation can be traced back to well before our current historic baselines [57], indicating that the proportion of original grounds lost is undoubtedly greater than indicated by our figures.

The lack of a pristine baseline in our data is reflected in the oyster size and density statistics. Early historic reports refer to oysters a foot long in the eighteenth to mid nineteenth centuries [57,58], however, the quantitative assessments of beds used in this study were conducted decades later, once evidence of overfishing of oyster reefs was already apparent [59]. That we found no significant difference in size over the time period examined is therefore unsurprising. Our national-level statistics for oyster density similarly did not show a significant decline over time, possibly also owing to over exploitation prior to our centennial baseline, in particular on the Atlantic coast [12] (figure 2*b*). Nevertheless, our results indicate that oyster grounds have declined markedly in condition over the time period examined, with biomass in some areas declining to a far greater extent than area. In fact since 1884, a number of historic reports have highlighted the inadequacy of using areal extent measures alone to determine oyster abundance and reef condition, observing that fishing activity often resulted in the expansion of oyster extent through the spreading out of shell, without necessarily increasing oyster abundance [58,59]. Indeed, this expansion probably reduced reef height [33], placing oysters in locations where their survival was reduced and therefore contributing to long-term losses of natural oyster reefs [60].

The declines in oyster ground extent and oyster biomass were not universal. The current oyster population in Apalachicola Bay, FL exceeds historic oyster abundance. This estuary represents one of the few estuaries in which fishing is primarily restricted to harvest by tongs (see §2), combined with intensive management and shell planting. Similarly, Sabine Lake, TX exceeds our historic estimates of abundance and has been closed to oyster fisheries for over 40 years. As our analysis includes only two time periods, we have no measure of whether change is still occurring and are therefore unable to assess whether our results are the product of current management or historic change.

While our data are useful in estimating the loss of ‘natural’ oyster grounds, a significant but unknown proportion of oysters in several regions in the US are located on leased grounds, notably eastern oysters in LA, the northeastern Atlantic coast, and on the west coast, where there is extensive aquaculture of the non-native *C. gigas* (Thunberg, 1793). We were unable to collate data on the extent of oyster habitat on leased grounds as these are rarely surveyed. This omission has limited impact on the importance of our findings as relates to natural oyster grounds, as many leased areas are heavily manipulated, with oysters often relocated several times before harvest. Leases may make a marked contribution to extent, biomass and ecosystem services from oysters, but these populations represent an extractable resource as opposed to habitat-forming reefs or beds. For areas such as LA, CT and NJ where leasing is extensive, our findings probably underestimate overall native oyster populations, but the comparisons of historic and present-day extent of natural oyster grounds remain valid. Another issue concerns oyster habitat created by wild populations of *C. gigas* on the west coast. Wild populations of this species are currently small or absent in our study estuaries, with the exception of Willapa Bay where the population is subject to rotational harvest, similar to other leased grounds [61]. Where populations of *C. gigas* occur, they may perform many of the ecological functions previously provided by native oysters [61].

In a recent analysis based on expert opinion and literature review, oyster reefs worldwide were estimated to have declined by 85 per cent, with the US faring relatively well [30]; thus, our more quantitative analysis of 64 per cent decline in extent of oyster grounds in the US appears at first glance to support our current understanding. However, as biomass losses were often more extreme than extent, the status of oysters appears more dire than indicated simply by area. This also has potential implications for estimates of ecosystem service delivery, as function may scale nonlinearly with both area and density [62]. Despite these documented declines, North America remains a region with some hope; stable or increasing oysters in some estuaries underscore that management and restoration efforts can be successful. Our centennial baselines provide a quantitative context to inform and motivate stakeholders, prioritize efforts and set goals for restoration, and ultimately bring these critical habitats back from the brink.

Our results represent, to our knowledge, the first effort to quantify both extent and biomass for a marine habitat-forming species across a centennial time period. Indeed, we believe that these findings may be unique at this large scale even among terrestrial studies. While many studies have provided compelling evidence of change in habitat extent over the last 100 years [16], and others have been able to compile localized or point source evidence of changes in abundance of certain species [63], few have been able to combine an assessment of change in extent and in biomass across large spatial and temporal scales.

These findings thus have a broader resonance for conservation biology generally. While change in extent remains a predominant metric in many analyses of human impact [2,18,52], our work confirms, with real numbers, that this may be insufficient for assessing overall changes to habitats. The altered and degraded condition of many present-day habitats can also lead to the undervaluing of their potential in terms of ecological function and ecosystem service provision. Improved historic baselines that take into account both extent and condition of habitats will greatly improve ongoing conservation planning, the relatively new science of ecosystem ‘red-listing’ [64]; and the ever-growing efforts to restore or rehabilitate lost and degraded habitats.
